# The Influence of Pre-IVF Day 2 TSH Levels on Treatment Success and Obstetric Outcomes: A Retrospective Single-Center Analysis with Machine Learning-Based Data Evaluation

**DOI:** 10.3390/jcm14134407

**Published:** 2025-06-20

**Authors:** Bernadett Nádasdi, Viktor Vedelek, Kristóf Bereczki, Mátyás Bukva, Zoltan Kozinszky, Rita Sinka, János Zádori, Anna Vágvölgyi

**Affiliations:** 1 Department of Medicine, Albert Szent-Györgyi Medical School, University of Szeged, 6725 Szeged, Hungary; 2 Department of Genetics, Faculty of Science and Informatics, University of Szeged, 6726 Szeged, Hungary; 3 Department of Obstetrics and Gynecology, Albert Szent-Györgyi Medical School, University of Szeged, 6725 Szeged, Hungary; 4 Department of Immunology, Albert Szent-Györgyi Medical School, Faculty of Science and Informatics, University of Szeged, 6720 Szeged, Hungary; 5 Department of Pediatrics and Pediatric Health Center, University of Szeged Albert Szent-Györgyi Health Center, Korányi Fasor 14-15, 6720 Szeged, Hungary; 6 Laboratory of Microscopic Image Analysis and Machine Learning, Institute of Biochemistry, Biological Research Centre, Hungarian Research Network (HUN-REN), 6726 Szeged, Hungary; 7 Capio Specialized Center for Gynecology, Solna, 17145 Stockholm, Sweden; 8 Institute of Reproductive Medicine, Albert Szent-Gyorgyi Medical School, University of Szeged, 6723 Szeged, Hungary

**Keywords:** TSH, body mass index (BMI), in vitro fertilization (IVF), clinical pregnancy, live birth

## Abstract

**Background:** Thyroid disorders, particularly thyroid autoimmunity, are increasingly prevalent among women of reproductive age and have been linked to fertility outcomes. While current endocrinology guidelines define distinct thyroid-stimulating hormone (TSH) target values for women undergoing assisted reproductive technology (ART), the optimal preconception TSH range for in vitro fertilization (IVF) success remains a topic of debate. **Objectives:** This study aimed to assess the impact of baseline TSH levels within the recommended normal range on IVF outcomes, specifically clinical pregnancy and live birth rates. Additionally, we assessed the predictive value of procedural and preprocedural factors, including maternal body mass index (BMI) and TSH, using machine learning models. **Methods:** We conducted a retrospective, single-center cohort study at the Institute of Reproductive Medicine, University of Szeged, involving 996 women who underwent IVF, with or without intracytoplasmic sperm injection. Biometric, medical history, laboratory, and procedural factors were analyzed. Pregnancy and live birth predictions were modeled using support vector machine (SVM), random forest (RF), and extreme gradient boosting (XGBoost) algorithms. The significance of features in the RF and XGBoost models was assessed. **Results:** SVM models achieved a mean accuracy of 72.26% in predicting pregnancy but were less effective for live birth classification. RF and XGBoost models demonstrated an area under the receiver operating characteristic curve of 0.76 and 0.74 for pregnancy and 0.67 and 0.61, respectively, for live birth. Key predictors included embryo score, maternal age, BMI, and specific hormone levels. Notably, male factors also contributed to outcome prediction. Analysis suggested that variations in maternal TSH within the normal range (0.3–4.0 mIU/L) had no significant impact on IVF success. **Conclusions:** Our study suggests that preconception TSH levels within the reference range do not significantly influence IVF success, which indirectly supports the validity of the current recommendations on this matter. While machine learning models demonstrated promising predictive performance, larger prospective studies are needed to refine thyroid function targets in ART, with a separate analysis of women with thyroid autoimmunity.

## 1. Introduction

Thyroid hormones play a crucial role in ovarian function, as evidenced by the presence of thyroid-stimulating hormone (TSH) and its receptors, along with thyroid hormone receptors (TR-α1 and β1), in the ovarian epithelium and oocytes across different follicular stages [1]. The expression of TSH receptor was also detected in human corpus luteum [2]. Thyroid hormones play an essential role in oocyte maturation and implantation; they can indirectly impact fertility by modulating GnRH and prolactin secretion, influencing sex hormone binding globulin (SHBG) levels, and affecting coagulation factors [3]. The prevalence of various thyroid disorders, recognized as influential chronic conditions, has been increasing among women of reproductive age, with thyroid autoimmunity, also known as Hashimoto’s thyroiditis, representing the most substantial proportion [4,5]. Consequently, thyroid function has become a central focus of research, particularly in its significant impact on fertility and the ongoing effort to define the optimal TSH range for reproductive potential. Multiple studies have reported that in women with subclinical and overt hypothyroidism, those receiving L-thyroxine treatment exhibited a significantly higher live birth rate and a significantly lower miscarriage rate compared to untreated controls [3,6,7]. Current endocrinology guidelines classify women undergoing assisted reproductive technology (ART) procedures into two distinct groups, each with specific TSH target values. For women without diagnosed thyroid disease, the recommended TSH target is below 4 mIU/L, aligning with reference values for the general healthy population. Additionally, treatment with L-thyroxine prior to ovarian stimulation is not advised for euthyroid women without thyroid autoimmunity. In contrast, for women with diagnosed thyroid disease, the recommended TSH target before assisted reproductive technology procedures is below 2.5 mIU/L, a reference range that has been globally endorsed [8,9]. In the study by Repelaer van Driel-Delprat [10], TSH levels between 2.5 and 4.5 mIU/L were not associated with differing fertility outcomes in the majority of women undergoing conventional in vitro fertilization (IVF). Furthermore, previous data suggest an increase in TSH in conjunction with body mass index (BMI) in obese subjects [11] and also in infertile women [12]. According to the recommendations of the National Institute for Health and Care Excellence (NICE), women should be advised to maintain a body mass index (BMI) within the range of 19–30 kg/m^2^ before starting assisted reproduction, as a BMI outside this range is associated with a lower success rate of ART procedures [13]. In our previous work [14], we extensively examined the impact of maternal BMI on in vitro fertilization (IVF) outcomes. Notably, follicle-stimulating hormone (FSH) and luteinizing hormone (LH) levels showed an unexpected decline as BMI increased, highlighting the evolutionary significance of nutritional status in reproductive function. However, TSH analysis was not performed, which may have limited our understanding of the potential role of thyroid function in reproductive outcomes and the influence of thyroid disorders on assisted reproduction success.

## 2. Goals

In our study, we aimed to assess the potential impact of baseline TSH levels within the aforementioned recommended normal range on in vitro fertilization outcomes by stratifying patients into quartiles. We also examined the relationship between medical history, preprocedural and procedural parameters, and IVF outcomes, specifically clinical pregnancy and live birth, with a particular focus on the role of baseline maternal BMI and TSH levels. Additionally, a support vector machine (SVM) model was developed to predict potential pregnancies based on medical data using 40 dimensions, while random forest (RF) and extreme gradient boosting (XGBoost) models were employed using the same 40 dimensions to identify the most relevant preprocedural and procedural factors influencing treatment outcomes. SVM model is one of the most studied methods; it is versatile and can handle high-dimensional data. In our previous work, we used SVM to classify implantation outcomes [14]. Additionally, we chose RF and XGBoost because these tree-based decision models showed high performance on fertility data [15].

## 3. Patients and Methods

### 3.1. Study Population

A retrospective, single-center cohort study was conducted at the Institute of Reproductive Medicine, University of Szeged. The data collection was performed from 21 January 2022 to 12 December 2023, among women treated for IVF with or without intracytoplasmic sperm injection (ICSI) with successful oocyte retrieval. The inclusion criterion was a verified baseline TSH value within the normal range of 0.3–4.0 mIU/L, as defined by Unuane and Velkeniers [3]. The exclusion criteria included patients with biochemical pregnancy, those with an ectopic pregnancy outcome, and those who did not meet the inclusion criteria. The patient population was not stratified based on levothyroxine therapy status, nor was the presence or absence of autoimmune thyroid disease assessed. A comprehensive set of biometric, medical history, laboratory, procedural, and paternal parameters, also presented in Table 1, was recorded and analyzed to evaluate factors influencing infertility treatment outcomes. Biometric data included age (years), weight (kg), height (cm), and BMI (kg/m^2^). Infertility related information covered the duration of infertility (years), previous births, miscarriages, and abortions, as well as the presence of Fallopian tube obstruction, prior unsuccessful intrauterine insemination (IUI), polycystic ovary syndrome (PCOS), endometriosis, paternal infertility or other causes of infertility (‘other indication’). Laboratory parameters encompassed baseline TSH (mIU/L), FSH (IU/L), LH (IU/L), and anti-Müllerian hormone (AMH; pmol/L). Procedural data included the duration of ovarian stimulation (days), the type of stimulation protocol (gonadotropin-releasing hormone [GnRH] agonist—Ultra Short/Short/Long or GnRH antagonist), the number of previous cycles and discontinued cycles, the number of follicles, the embryo score of the best transferred embryo, and endometrial thickness measurements on the day of triggering human choriogonadotropin (hCG) injection (ENDOV; mm), at follicular puncture (ENDPU; mm), and at embryo transfer (ENDET; mm). Additionally, paternal factors were evaluated in accordance with the recent WHO guidelines [16], including paternal age (years) and semen analysis parameters such as sperm concentration (×10^6^/mL), normal sperm motility (%), and the prevalence of oligospermia (*n*; %), asthenozoospermia (*n*; %), teratozoospermia (*n*; %), and normospermia (*n*; %). These parameters were systematically recorded and analyzed to assess their potential impact on fertility treatment success. In this study, the primary outcomes assessed were clinical pregnancy and live birth. Clinical pregnancy was determined at 7 weeks of gestational age, following the recommendations of the International Committee for Monitoring Assisted Reproductive Technology [17]. It was identified through the visualization of one or more gestational sacs (excluding ectopic pregnancies) using transvaginal ultrasound.

### 3.2. Methods

Ovarian stimulation was carried out using patient-tailored flexible GnRH agonist or antagonist protocols (Table 1), adjusted based on the women’s ovarian function, sex hormone profile, age, and body weight. Cycle selection took place on day 2 or 3, during which an initial assessment was conducted. This included ultrasound examination (Samsung Medison HS50; endocavitary probe: EVN4-9, 4–9 MHz), estimation of antral follicle count, and evaluation of FSH, LH, prolactin, and TSH. The GnRH treatment protocol, as well as laboratory and embryo transfer procedures, remained consistent across all cycles and adhered to standard laboratory guidelines. A detailed description of these protocols can be found in our previous publication [14] (Supplementary Materials).

Endometrial thickness was measured using the same ultrasound device on three occasions: on the day of hCG trigger injection (ENDOV; mm), at the time of oocyte retrieval (ENDPU; mm), and at embryo transfer (ENDET; mm). Measurements were taken from the outer edge of the endometrial-myometrial interface to the outer edge at the widest part of the endometrium [18].

To account for differences in embryo development stage and quality on various transfer days, an individual evaluation system was implemented. For statistical analysis, a specific embryo point score [19,20,21] was used, with the corresponding details provided in Supplementary Materials (Supplementary Table S1).

### 3.3. Statistical Analysis

For general data management, Microsoft Excel was used. Data analysis and exploration were conducted using Jupyter Notebook version 6.3.0. Data handling and statistical analyses were performed with Python 3.6, utilizing the NumPy (1.22.4), Pandas (1.2.4), SciPy (1.6.2), XGBoost (2.1.3), Sensitivity (0.2.8), SHAP (0.44.1), and scikit-learn (1.3.2) libraries. For visualization, the Matplotlib (3.3.4) and Seaborn (0.11.1) libraries were utilized. Smooth curve fitting and generalized additive models were implemented in R 4.4.3 using the gam function from the mgcv library (1.9-3) [22].

To assess statistical significance between quartiles, ANOVA was performed. *p*-values below 0.05 were considered significant.

Correlation was measured using the Pearson coefficient, and the corresponding two-sided *p*-values were calculated with the SciPy function scipy.stats.pearsonr.

In order to make outcome predictions from the data, classification algorithms were used. For classification purposes, we chose to test three different machine learning methods. Support vector machine (SVM) and random forest classifier (RF) models were developed with scikit-learn, and extreme gradient boosting (XGBoost) models were constructed using the XGBoost library. We listed the main parameters and conditions we used for model testing and optimization. SVM models were iterated through 50 random states, and grid optimization was applied in every case (C: 0.1, 1, 10, 100, 1000; gamma: 1, 0.1, 0.01, 0.001, 0.0001, 0.00001, 0.000001). RF models were optimized in 30 iterations and 5-fold cross validation using randomized grid (estimators: 100, 307, 514, 721, 928, 1135, 1342, 1550, 1757, 1964, 2171, 2378, 2585, 2792, 3000; max depth: 1, 5, 10, 20, 50, 75, 100, 150, 200; minimum samples split: 1, 2, 5, 10, 15, 20, 30; minimum samples leaf: 1, 2, 3, 4; max features: auto/square root; bootstrap: yes/no; criterion: gini/entropy), then optimized using grid search with 5-fold cross validation (estimators: between 1600 and 1900 by 5; max depth: 130, 140, 150, 160, 170; minimum samples split: 2, 3, 4; minimum samples leaf: 2, 3, 4, 5; bootstrap: yes; criterion: entropy). The XGBoost models underwent 10-fold cross-validation with 250 iterations, using Bayesian optimization (maximum depth: 2–8; learning rate: 0.001–1; subsample: 0.5–1; column sampled by tree, level, and node: 0.5–1; alpha: 0–10; lambda: 0–10; gamma: 0–10).

Data preprocessing included the use of the Synthetic Minority Oversampling Technique (SMOTE) to oversample the minority class and prevent overfitting, as well as basic data standardization. Data were randomly split, with 80% used for training and 20% for testing in RF and XGBoost, and a 70–30% split in SVM.

## 4. Results

**Figure 1:**
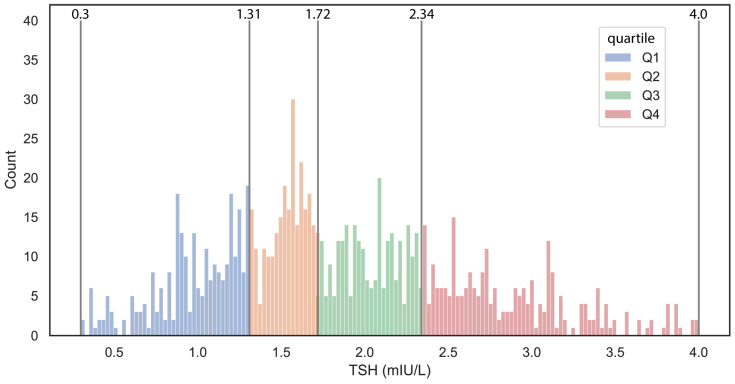
Distribution of the TSH levels in the cohort. The histogram illustrates the distribution of TSH levels, with distinct colors assigned to each quartile. The visualization makes visible not just the distribution of values but the range of each quartile. The minimum and maximum values of the quartiles are marked with gray vertical lines, which also display the associated values.

During the aforementioned time interval, 1086 women with a registered TSH level underwent in vitro fertilization and embryo transfer treatment at the Institute of Reproductive Medicine, University of Szeged. Eighteen patients were excluded due to ectopic pregnancy, and an additional 22 subjects were excluded due to missed abortion, as these cases could not be categorized as either non-pregnant or clinically pregnant. Only subjects with a confirmed normal baseline TSH value within the 0.3–4.0 mIU/L range were included in the analysis, resulting in the exclusion of an additional 50 subjects. As a result, data from a total of 996 subjects were analyzed. The overall IVF-treated population was categorized into TSH interquartile groups as follows: Quartile I ranged from 0.3 to 1.31 mIU/L (*n* = 255), Quartile II from 1.32 to 1.72 mIU/L (*n* = 247), Quartile III from 1.73 to 2.34 mIU/L (*n* = 247), and Quartile IV from 2.35 to 4.0 mIU/L (*n* = 247). In the TSH distribution (Figure 1), the first three quartiles, covered a range of 0.3 to 2.34 mIU/L, whereas the upper quartile has a lower boundary close to the debated upper TSH reference limit of 2.5 mIU/L for women with subfertility. Among the quartiles, a weak significant difference was observed only for LH between the groups, while no significant differences were found for other examined parameters. Notably, there was no significant difference in outcomes, namely clinical pregnancy and live birth, either. We investigated the effect of element size and tested the differences between quartiles using a subset of random 300 and 500 patients’ data. Increasing the element number, the significant changes were decreasing, strengthening previous observations (Supplementary Table S2).

**Figure 2:**
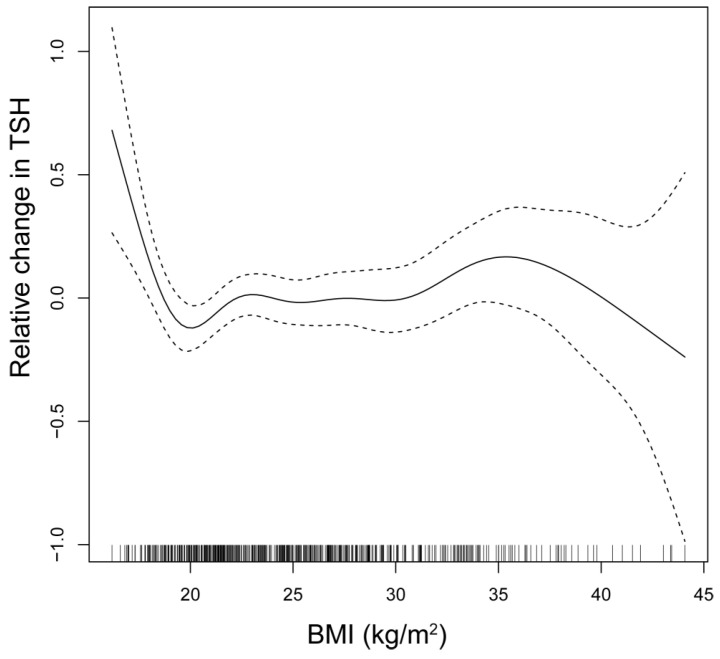
The relationship between BMI and TSH, modeled by smooth curve fitting. The solid line represents the smooth function estimates, the relative changes of TSH levels as a function of the BMI. The dashed lines represent 95% confidence intervals; where dashed lines are close to the solid line, the confidence is higher, and where they are more distant, the confidence is lower. The rug plot above the *x*-axis shows the distribution of BMI data; the representation of data points helps to interpret the low confidence zones. BMI, body mass index; TSH, thyroid-stimulating hormone.

The relationship between TSH and BMI is depicted through smooth spline analysis (Figure 2). The figure reveals that in regions of high confidence, TSH levels show minimal fluctuations, although a marginal increase in TSH levels can be observed with an increase in BMI. However, BMI does not change significantly between the TSH quartiles.

**Figure 3:**
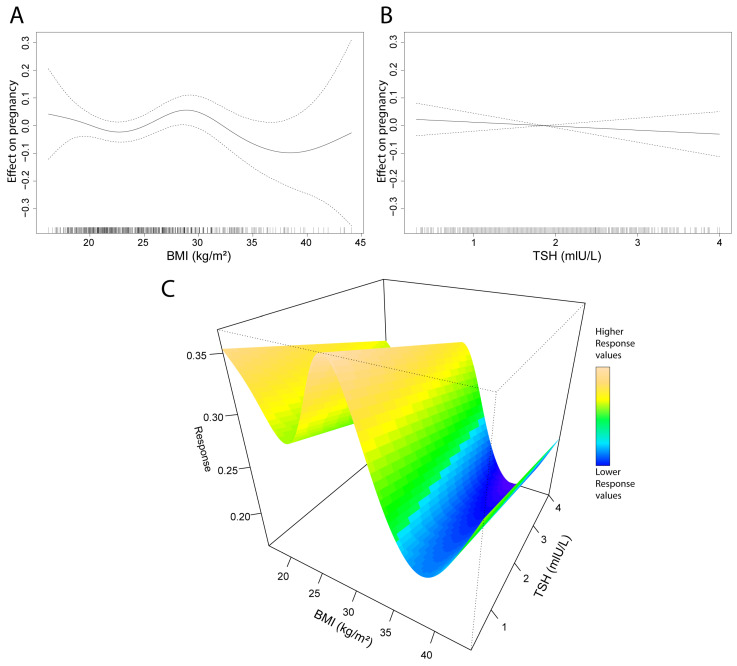
The relationship between pregnancy, BMI, and TSH, modeled by smooth curve fitting. (**A**,**B**) The solid line represents the smooth (**A**) and linear (**B**) function estimates, showing pregnancy as a function of BMI (**A**) and TSH (**B**). The dashed lines represent 95% confidence intervals; where dashed lines are close to the solid line, the confidence is higher, and where they are more distant, the confidence is lower. The rug plot above the *x*-axis shows the distribution of (**A**) BMI and (**B**) TSH data, and the representation of data points helps to interpret the low confidence zones. (**C**) A 3D representation of the GAM model of BMI and TSH effects on pregnancy; the visualization consists of the components of A and B together, without confidence intervals. BMI, body mass index; TSH, thyroid-stimulating hormone.

Generalized additive model (GAM) analyses were conducted on pregnancy, BMI, and TSH data. Neither parameter shows a strong association with pregnancy outcomes; however, compared to BMI, the impact of TSH appears even more negligible (Figure 3A,B). Interestingly, the BMI smooth spline reveals a peak in the BMI range of 27–30. The TSH GAM produces a linear model, indicating that reduced levels of TSH have a very limited positive impact on pregnancy outcomes. Investigating the effect of both BMI and TSH shows similar results (Figure 3C). Additionally, we tested the TSH levels in age and BMI groups to see potential significant differences, but we found none (Supplementary Figure S1A). Also, sensitivity analyses were performed, showing higher differences at the extreme values, in accordance with the GAM model (Supplementary Figure S1B).

### 4.1. Correlations in the Whole Population

In the correlation analysis, TSH did not show a significant correlation with any of the examined parameters. Our other index dimension, BMI, showed a correlation only with LH (r = −0.2; ***) among all the examined parameters. The results of the correlation analysis can be found in the Supplementary Table S3, and their heatmap representations are shown in Supplementary Figure S2.

### 4.2. Pregnant Versus Non-Pregnant Subgroup Analysis

In the studied population, 273 individuals achieved clinical pregnancy; these participants will be referred to as the “pregnant group,” while the remaining 723, who did not conceive, will be designated as the “non-pregnant group”. When comparing the two groups, the pregnant individuals were younger (33.9 ± 4.31 vs. 36.5 ± 5.05 years; ***), had a significantly lower number of cycles (1.8 ± 1.06 vs. 2.1 ± 1.42 IU/L; ***), lower FSH levels (7.72 ± 3.08 vs. 8.19 ± 3.40 IU/L; ***), higher AMH levels (3.1 ± 2.60 vs. 2.3 ± 2.21 pmol/L; ***), and a shorter duration of infertility (3.8 ± 2.69 vs. 4.4 ± 3.01 years; ***). In the pregnant group, the number of follicles was significantly higher (8.9 ± 3.89 vs. 7.5 ± 3.68; ***), and the embryo score was notably better for both the first (2.7 ± 0.51 vs. 2.3 ± 0.71; ***) and the second embryo (2.2 ± 0.68 vs. 1.9 ± 0.75; ***). Paternal age was also significantly lower in the pregnant group (37.0 ± 5.59 vs. 38.9 ± 6.27 years; ***), while sperm concentration (50.5 ± 40.60 vs. 45.1 ± 40.88 × 10^6^/mL; *) and sperm motility (45.3 ± 18.97 vs. 42.0 ± 18.52%; *) were both significantly higher. Between the pregnant and non-pregnant groups, no significant difference was observed in the two examined index dimensions, TSH (1.9 ± 0.72 vs. 1.9 ± 0.76 mIU/L) and BMI (24.9 ± 5.11 vs. 25.2 ± 5.26 kg), respectively.

### 4.3. BMI19-30 Versus BMI-Out Subgroup Analysis

We examined the population based on the recommended target BMI before IVF according to the NICE recommendation, comparing individuals with a BMI between 19 and 30 kg/m^2^ (BMI19-30; *n* = 739) and those outside this range (BMI-Out; *n* = 257). A significant difference between the two groups (BMI19-30 vs. BMI-Out, respectively) was found in maternal age (36.0 ± 4.90 vs. 35.2 ± 5.23 years, *), TSH levels (1.8 ± 0.74 vs. 2.0 ± 0.77 mIU/L; *), FSH levels (8.2 ± 3.54 vs. 7.2 ± 2.59 IU/L, ***), LH levels (6.1 ± 2.78 vs. 5.5 ± 3.06 IU/L, **), and AMH levels (2.4 ± 2.33 vs. 2.7 ± 2.40 pmol/L, **). Regarding anamnesis data, the duration of infertility was significantly lower in the BMI19-30 group (4.1 ± 2.76 vs. 4.7 ± 3.37 years, *). In terms of procedural factors, both ENDPU and ENDET were significantly thinner in the BMI19-30 group (10.5 ± 1.95 vs. 10.8 ± 2.33 mm, *; 11.4 ± 2.44 vs. 11.92 ± 2.51 mm, **, respectively). Although the embryo score of the first embryo was significantly higher in the BMI19-30 group (2.5 ± 0.68 vs. 2.3 ± 0.72, *), no significant advantage was observed in clinical pregnancy and live birth rates.

### 4.4. Support Vector Machine, Random Forest Classification, and Extreme Gradient Boosting

We were interested in determining whether the current dataset could be used for classifying the pregnant and non-pregnant groups, as well as predicting live births. Moreover, we were interested in the potential effect of TSH on the models. One of the most versatile and commonly used classification algorithms is the support vector machine. This method is capable of handling high-dimensional data. The method investigates the boundary between the defined groups of a training set using vectors. We used support vector machine models as previously described [14] to assess potentially interesting predictors. We checked the model’s performance on the entire dataset and iterated through a collection of models in order to identify differences when a dimension is missing.

**Figure 4:**
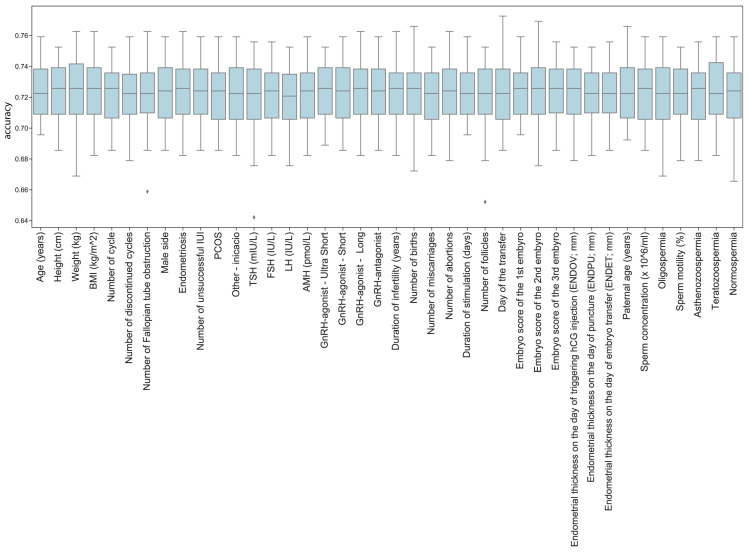
The boxplot represents the accuracy of the SVM model iterations. Minimum and maximum values are represented at the bottom and top of each category. Lowest and highest quartiles are represented as lines, the middle quartiles as boxes, and the median value is represented by a line within each box. Outliers are represented as diamonds. Features omitted are displayed on the *x*-axis.

The models reached a mean accuracy of 72.26% in predicting pregnancy, which represents a considerable improvement compared to previous results. However, the method was not effective in testing the effect of different features with the current dataset, including TSH (Figure 4).

**Figure 5:**
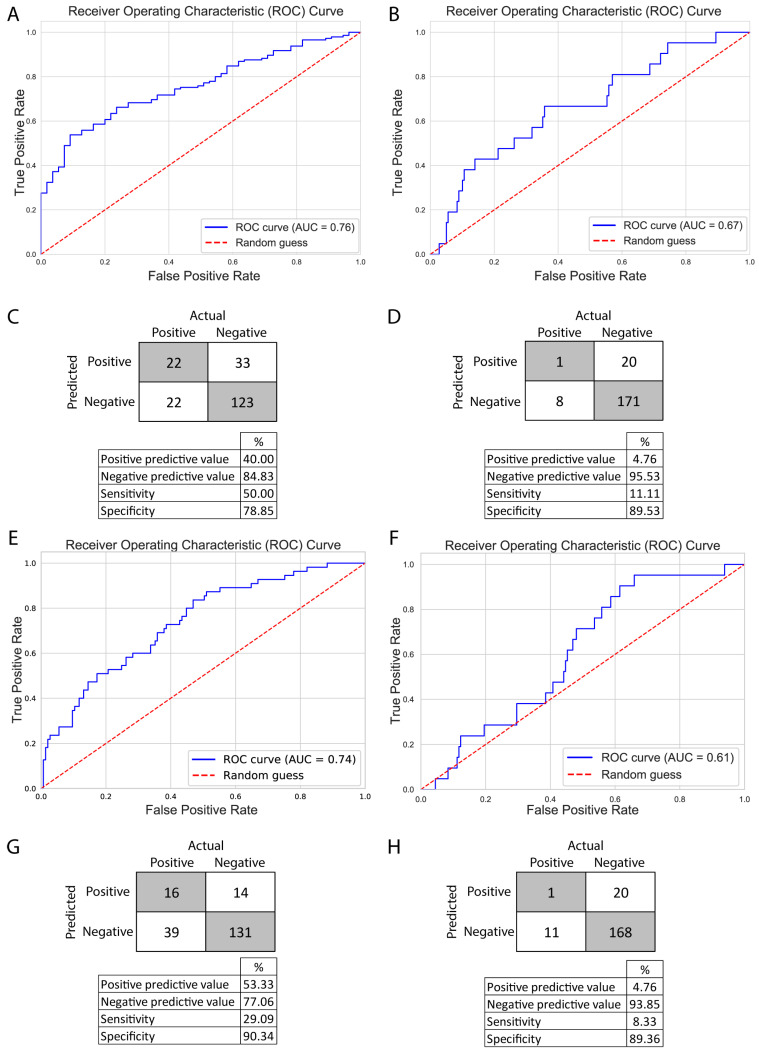
RF and XGBoost model performances. RF: (**A**,**C**) represent metrics for the classification of pregnancy. (**B**,**D**) represent metrics for the classification of live birth. (**A**,**B**): ROC/AUC analysis of the models. (**C**,**D**): Confusion matrices and performance data of the corresponding models. XGBoost: (**E**,**G**) represent metrics for the classification of pregnancy. (**F**,**H**) represent metrics for the classification of live birth. (**E**,**F**): ROC/AUC analysis of the models. (**G**,**H**): Confusion matrices and performance data of the corresponding models. ROC/AUC graphs represent a random guess as a red dotted line; if a model is random guessing between two categories (50/50%), the curve would fit on this line. On the *y*-axis, we can observe the ratio of true positive hits (values between 0 and 1); the optimal values here are close to 1, as we wish to be as accurate as possible. The *x*-axis represents the false positive ratio (0–1); ideally, this value should be close to 0. A perfect model would draw a square where there are no false positive values and only true positive values are present; in this case, the area under the curve would be 1. In the case of a random guess, the area under the curve is 0.5. Better models have values closer to 1. Confusion matrices show the categorical results of the classified test data. True positives and negatives are categories where the model classified correctly, and the labels are the same. False positives and negatives are when the actual and predicted labels are different. ROC, receiver operator curve; AUC, area under the curve.

Therefore, we explored a different approach: two decision tree-based methods, random forest (RF) and extreme gradient boosting (XGBoost), as these methods produce feature importance metrics. In the case of RF, feature importance metrics are computed as the mean and standard deviation of the accumulation of the impurity decrease within each tree. Meanwhile, XGBoost allowed us to gain insight into three different aspects: gain (average gain on splits), weight (how often the feature was used), and cover (average coverage of splits). We constructed two models with both methods, one to predict pregnancies and one to predict live births. Due to the underrepresentation of pregnancies and live births in the dataset, we employed the Synthetic Minority Oversampling Technique (SMOTE) before constructing the models to prevent overfitting. The RF models were grid-search optimized, and the XGBoost models were subjected to a 10-fold cross-validation over 250 iterations, employing Bayesian optimization techniques. The optimized models were evaluated using receiver operator curve—area under the curve (ROC-AUC), achieving an AUC of 0.76 for pregnancy and 0.67 for live birth with RF and an AUC of 0.74 for pregnancy and 0.61 for live birth with XGBoost. Models for pregnancy and live birth showed a relatively good specificity (RF: 78.85%, 89.53%; XGBoost: 90.34%, 89.36%) and negative predictive values (RF: 84.83%, 95.53%; XGBoost: 77.06%, 93.85), meanwhile, the models’ sensitivity (RF: 50%, 11.11%; XGBoost: 29.09%, 8.33%) and positive predictive values (RF: 40%, 4.76%; XGBoost: 53.33%, 4.76%) were weak. Although the models predicting live births successfully identified negative outcomes, they faced challenges in classifying positive outcomes (Figure 5).

While the models performed relatively well in classifying pregnancies (AUC 0.76 and 0.74), they were not effective in predicting live births (AUC 0.67 and 0.61). This may be explained by the lower number of live birth cases, suggesting that model performance could improve with additional data. Comparing RF and XGBoost, we observed better and more balanced scores with RF, but the positive predictive value was better with the XGBoost model.

**Figure 6:**
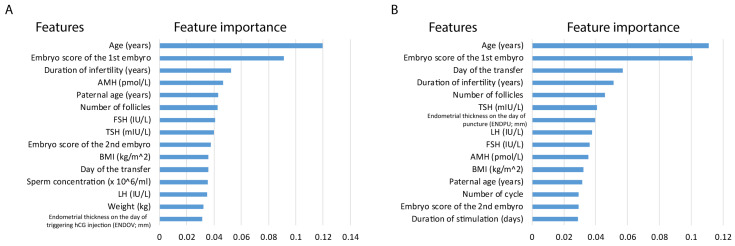
Top 15 features of RF models. (**A**) Feature importance associated with the classification model for clinical pregnancy. (**B**) Feature importance associated with the classification model for live births.

Despite the performance differences between pregnancy and live births, by investigating the top 15 feature importance of the models, we observed that similar features played a role in the analyses. Feature importance in the live birth model is not conclusive as the model underperforms; yet it can give insights in light of the better-performing pregnancy model. In RF models, the majority of the top features are shared, including age (years), paternal age (years), embryo score of the 1st embryo, LH (IU/L), AMH (pmol/L), TSH (mIU/L), FSH (IU/L), BMI (kg/m^2^), day of the transfer, number of follicles and duration of infertility (years). It is important to highlight age and embryo score as high-impact features in both analyses. Moreover, TSH also appeared in both models (Figure 6).

**Figure 7:**
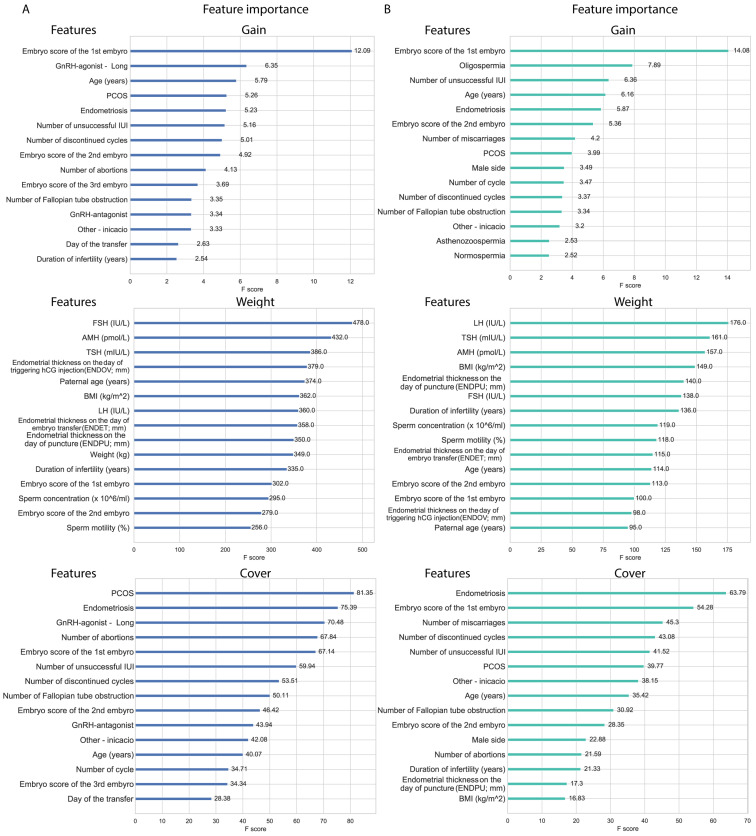
Top 15 features of XGBoost models in gain, weight, and cover. (**A**) Feature importance associated with the classification model for clinical pregnancy. (**B**) Feature importance associated with the classification model for live births.

XGBoost models’ feature importance in gain ranking highlights features that improve the model accuracy; in this aspect, the embryo score of the first embryo stands out in both pregnancy and live birth models. Alongside embryo score, age and endometriosis are also in the top five features in both models. In the top 15 features, PCOS, number of unsuccessful IUIs, the number of discontinued cycles, the embryo score of the second embryo, the number of fallopian tube obstructions, and ‘other indication’ were shared between the two models, suggesting the relevance of these features. Features in weight (or frequency) represent how often a given feature was used. The features represented in weight are mostly non-categorical features with a normal distribution, including hormone (LH, FSH, TSH) levels, endometrial data, weight, BMI, age, and spermiogram data. Both models share AMH and TSH in the top five features, and share FSH, LH, duration of infertility, the three endometrial data, embryo scores of the first and second embryo, sperm concentration and motility, and maternal and paternal age in the top 15 features. Cover represents the average coverage across all splits where the feature is used. In cover, the models also share two features in their top five features, which are endometriosis and the embryo score of the first embryo, but they share features in the top 15 as well; PCOS, number of discontinued cycles, number of abortions, number of fallopian tube obstruction, number of unsuccessful IUIs, embryo score of the second embryo, ‘other indication’, and age. Features observed in both weight and gain also appeared in the cover data. Notably, male factors also appeared as predictors in both models (Figure 7).

**Figure 8:**
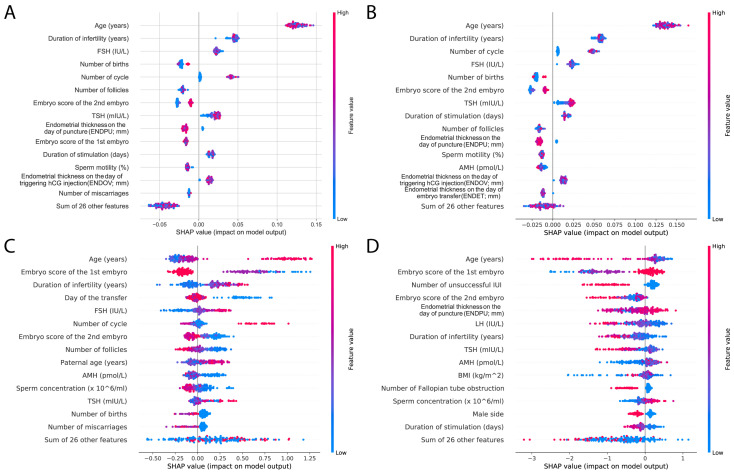
Top 15 features of RF and XGBoost models using SHAP analysis. Beeswarm plots represent SHAP analyses of RF (**A**,**B**), XGBoost (**C**,**D**) models of pregnancy (**A**,**C**), and live birth (**B**,**D**). The beeswarm plot illustrates how the top features in a dataset impact the model’s output. For each instance, the corresponding explanation is symbolized by a single dot on each feature row. The *x*-axis represents the SHAP value. The accumulation of dots along each feature row shows the density. Color is used to display the original value of a feature.

In order to compare different models, we used SHapley Additive exPlanations (SHAP) analysis [23]. Again, we investigated the top 15 features; similar features appeared in the top 15 results as previously. The most important factor was age in all of the models; however, hormone levels, including TSH, FSH, and AMH, were also present. Duration of infertility and embryo scores were also represented, as well as endometrium data. The SHAP results also highlight the difference between the RF and XGBoost models: RF SHAP values are more distinct and clustered together, whereas the SHAP values of XGBoost models are more scattered (Figure 8).

RF and XGBoost models share the embryo score as a high-impact feature, but surprisingly, in XGBoost, age has a smaller gain. Although the SHAP analyses showed that age also plays an important role in XGBoost models, this might nevertheless explain the better AUC scores of the RF model. Also, hormone levels and BMI show little gain in XGBoost models, but are present in weight features; meanwhile, these features are in the top 15 of RF models. Interestingly, TSH as a feature shows similar effects to other hormones.

## 5. Discussion

The population presented based on TSH quartiles, examined across 43 dimensions, did not show significant differences between the groups. Only a weak significant difference was observed in LH levels, which were significantly higher in Q3 compared to the values in the other quartiles (Table 1). The different TSH ranges appear to be indifferent regarding the other examined parameters of the population. No differences were observed between the quartiles in terms of clinical pregnancy and live birth, further indicating that baseline TSH levels within the recommended range before IVF have a similar impact on the success of these outcomes. This is further supported by the results of the correlation analysis conducted on the entire population, in which TSH did not show a significant correlation with any of the examined parameters. In the pregnant versus non-pregnant subgroup analysis, no significant difference in TSH levels was observed between the two groups.

Our study emphasizes that preconception TSH levels within the normal range have no substantial impact on IVF success, particularly in terms of clinical pregnancy or live birth, indirectly reinforcing the credibility of existing guidelines on this topic. A similar conclusion was reached by Coussa et al. [24], who found that variations in maternal TSH levels within the normal range (0.4–4.0 μIU/mL) before conception do not influence the success of IVF treatment or pregnancy outcomes. Their findings do not support the recommendation of maintaining preconception TSH levels at or below 2.5 μIU/mL for pregnancies conceived through IVF, but rather promote a preconception TSH level within the normal range. Mintziori et al. [25] aimed to assess the relationship between TSH concentrations and the presence of thyroid autoimmunity with live birth rates in euthyroid women undergoing IVF. They found no difference in live birth rates between the subgroups of euthyroid women with TSH levels of 0.5–2.5 μIU/mL versus 2.6–4.5 μIU/mL, nor between those with or without thyroid autoimmunity. Although our study did not investigate the presence of thyroid autoimmunity, these findings support the notion that TSH levels within the euthyroid range do not adversely affect IVF reproductive outcomes. The study by d’Assunção et al. [26] found that variations in TSH levels within the normal range were not linked to pregnancy or delivery rates in women without autoimmune thyroid disease undergoing IVF treatment.

In addition to TSH, our focus also centered on BMI. In the analysis based on TSH quartiles, no significant difference was observed in maternal BMI among the groups. According to NICE recommendations, women should aim to maintain a BMI within the range of 19–30 kg/m^2^ before starting assisted reproduction, as a BMI outside this range is associated with a lower success rate in ART procedures [13]. Based on this, we divided the studied population into two groups: those within the target BMI range (BMI19–30) and those outside it (BMI-Out), and conducted a comparative analysis. While no significant difference was observed between the two groups in terms of clinical pregnancy and live birth rates, the BMI-Out group exhibited higher AMH levels, which were associated with better IVF success rates [27]. It is noteworthy that there was no significant difference in the prevalence of PCOS between the two groups. However, the BMI19–30 group had significantly thinner endometrial thickness at the time of oocyte retrieval and embryo transfer. Additionally, maternal age was significantly higher, and their baseline FSH and LH levels were also elevated compared to the BMI-Out group. These factors may contribute to diminishing the advantages typically associated with a favorable BMI. Additionally, TSH was significantly lower in the BMI19–30 group. In the present analysis, given the available sample size, we could not clearly confirm the advantage of the NICE recommendation; however, no disadvantage was observed regarding IVF outcomes either. Although the sample size was adequate in the present analysis, further analysis is planned with an increased sample size to investigate this aspect.

Smooth spline alignment on pregnancy outcomes and BMI showed a weak connection between the parameters. Yet, a peak was observed approximately at BMI 29, and a drop in the higher BMI range. These data suggest that moderately high BMI is not necessarily disadvantageous for pregnancy; however, after this peak, the negative implications of a higher BMI obviously manifest. Nevertheless, this could be specific to the investigated population; therefore, the expansion of sample size could also give more insights into this phenomenon.

SVM machine learning models performed significantly better with the current dataset compared to our previous investigation [14]. Yet, RF and XGBoost models provided more detailed insights into the data through feature importance and SHAP analysis. Previously, a study by Amini et al. (2021) showed that RF models perform better than XGBoost models in live birth predictions [15]. We obtained similar results, but while the RF model showed a better overall score, the XGBoost model had better positive predictive values. Although TSH does not considerably enhance the XGBoost model’s performance (gain) or coverage, it is favored in terms of weight. In RF, it is also present in the top 15 features. This indicates that the models regularly use TSH as a feature for their splitting decisions, in a similar manner to other hormone levels. The importance of TSH was reinforced in the SHAP analysis. This might be a random effect, but it also raises the possibility of special cases where TSH levels provide additional importance to the models. Despite the considerable performance gap between the classification models for pregnancy and live birth, the models utilized very similar features. The increasing amount of live birth data is expected to enhance model performance. Interestingly, in XGBoost, differences emerged between the investigated features: gain and cover-based feature importance highlighted embryo quality, age, endometriosis, and PCOS, while weight-based feature importance primarily reflected hormonal and endometrial factors.

In recent decades, ART has rapidly developed and has taken an increasingly important role in childbearing in developed societies: for infertile couples, ART represents a real alternative in family planning. However, despite this progress, its application still carries numerous risks, including possible complications such as ovarian hyperstimulation syndrome, gestational diabetes, pregnancy-induced hypertension, pregnancy with twins and triplets, preterm birth, low birth weight, and small gestational size [28]. Beyond the physical effects, we must also consider the psychological challenges experienced during the treatments, not to mention the emotional burden that couples may face in the event of unsuccessful therapy. Taking these factors and the high financial costs into account, there is an ongoing need to determine a realistic probability of treatment success before starting the procedure, so that couples can make responsible decisions before undergoing treatment. The emergence of artificial intelligence (AI) and machine learning (ML) in medicine has made it possible to analyze various influencing factors, enabling a more accurate outcome prediction for reproductive treatments [29]. In both embryology and andrology, ML techniques have already achieved substantial integration, facilitating advancements such as automated embryo grading and selection for transfer, as well as the development of intelligent sperm selection technologies for fertilization [30]. ML methods can also play a significant role in reproductive medicine across additional domains, including the personalized optimization and selection of treatments, as well as the more precise evaluation of treatment outcomes—areas that were central to our investigation. While the majority of AI algorithms and computational methods may be difficult to implement, there are now many open-source and free software libraries available that are capable of accurate data integration and analysis, continuous learning, and managing nonlinear and complex relationships [31]. According to current literature, a wide variety of ML techniques are increasingly contributing to a more nuanced understanding of how BMI and TSH affect IVF outcomes [32,33,34,35,36]. In these studies, BMI was a key predictor [32,33,34]. Interestingly, in our data, BMI was not a main predictor, and the negative effect of high BMI was mainly detected with the GAM analysis. It is important to notice that, next to different ML approaches and sample sizes, these studies are conducted on different populations or subpopulations as well. Regarding TSH, only a limited number of studies are available in the context of ART [35,36]. The predictive value of TSH was reported, which is in accordance with our results. Despite the fact that we could not detect significant differences with our univariate analysis, TSH levels were utilized as a predictor in different models, suggesting their importance in certain cases.

## 6. Conclusions

Our study highlights that preconception TSH levels within the normal range do not significantly impact IVF success, including clinical pregnancy and live birth rates. No significant differences were observed between the quartiles in terms of clinical pregnancy and live birth rates, further suggesting that baseline TSH levels within the recommended range prior to IVF exert a similar influence on these outcomes.

Additionally, while BMI within the recommended range (19–30 kg/m^2^) was associated with better embryo score of the first embryo, other confounding factors, such as maternal age and hormonal variations, may influence its effect on IVF outcomes.

Depending on the applied methodology, different aspects of medical history, preprocedural, and procedural factors may come into focus to varying degrees. Machine learning models demonstrated strong predictive potential, with RF and XGBoost providing deeper insights into key determinants of IVF success. Differences in feature importance suggest that embryo quality, age, and specific reproductive conditions play a pivotal role in live birth prediction. As more live birth data become available, model performance is expected to improve, enhancing personalized predictive capabilities for assisted reproduction outcomes.

Our results reinforce the multifactorial nature of IVF success. Artificial intelligence-supported predictive models have the potential to enhance personalized outcome predictions in reproductive medicine. Preprocedural estimations can aid couples in making informed decisions and provide psychological support by offering a realistic outlook on their reproductive potential. Furthermore, these estimations, when further refined by procedural data, could prove invaluable in guiding both infertile couples and their medical teams.

### Limitations

The primary limitation of this study stems from its retrospective design. Expanding the sample size may facilitate the identification and validation of more nuanced associations. Furthermore, our analysis does not consider potential thyroid disorders, thyroid autoimmunity, the effects of thyroid hormone substitution, or free thyroid hormone levels, such as thyroxine (T4) and triiodothyronine (T3), all of which represent important factors warranting further investigation in future research. Additionally, another limitation is the lack of testing the models on an independent cohort, which would provide useful information about the generalizability of these models.

## Figures and Tables

**Table 1:** Relevant clinical data in the study groups.

Clinical Data	Q1 [0.3–1.31] (*n* = 255)	Q2 [1.32–1.72] (*n* = 247)	Q3 [1.73–2.34] (*n* = 247)	Q4 [2.35–4.0] (*n* = 247)	Sig.
**Biometric Data**					
Maternal age (years)	35.8 ± 5.08	35.6 ± 4.86	36.0 ± 4.94	35.7 ± 5.12	n.s.
Height (cm)	165.5 ± 6.55	166.1 ± 6.36	165.8 ± 6.52	165.9 ± 6.09	n.s.
Weight (kg)	67.6 ± 14.81	70.3 ± 14.78	68.4 ± 15.26	69.8 ± 14.95	n.s.
BMI (kg/m^2^)	24.6 ± 4.94	25.5 ± 5.36	24.8 ± 5.10	25.4 ± 5.39	n.s.
**Preprocedural Data**					
Duration of infertility (years)	4.1 ± 2.90	4.2 ± 2.99	4.6 ± 3.01	4.1 ± 2.85	n.s.
Previous births (*n*; %)	55 (22)	53 (21)	38 (15)	44 (18)	n.s.
Previous miscarriages (*n*; %)	44 (17)	32 (13)	46 (19)	34 (14)	n.s.
Previous abortions (*n*; %)	30 (12)	26 (11)	39 (16)	35 (14)	n.s.
Fallopian tube obstruction (*n*; %)	70 (27)	77 (31)	71 (29)	56 (23)	n.s.
Unsuccessful IUI (*n*; %)	79 (31)	67 (27)	63 (26)	65 (26)	n.s.
PCOS (*n*; %)	25 (10)	20 (8)	16 (6)	17 (7)	n.s.
Endometriosis (*n*; %)	21 (8)	13 (5)	25 (10)	26 (11)	n.s.
Male side infertility (*n*; %)	115 (45)	116 (47)	115 (47)	103 (42)	n.s.
TSH (mIU/L)	1.0 ± 0.26	1.6 ± 0.11	2.0 ± 0.18	2.9 ± 0.41	***
FSH (IU/L)	7.6 ± 2.83	7.7 ± 3.38	8.1 ± 3.26	8.2 ± 3.82	n.s.
LH (IU/L)	5.9 ± 3.22	5.7 ± 2.20	6.5 ± 3.30	5.8 ± 2.57	*
AMH (pmol/L)	2.7 ± 2.48	2.3 ± 1.71	2.4 ± 2.32	2.6 ± 2.74	n.s.
**Procedural Data**					
Duration of stimulation (day)	10.5 ± 2.32	10.27 ± 1.75	10.2 ± 1.92	10.2 ± 1.88	n.s.
GnRH-agonist—Ultra Short (*n*; %)	1 (0)	3 (1)	1 (0)	0 (0)	n.s.
GnRH-agonist—Short (*n*; %)	115 (45)	116 (47)	115 (47)	103 (42)	n.s.
GnRH-agonist—Long (*n*; %)	21 (8)	13 (5)	25 (10)	26 (11)	n.s.
GnRH-antagonist (*n*; %)	79 (31)	67 (27)	63 (26)	65 (26)	n.s.
Number of cycles	2.0 ± 1.43	2.0 ± 1.23	2.2 ± 1.45	2.0 ± 1.20	n.s.
Number of discontinued cycles	0.1 ± 0.44	0.2 ± 0.44	0.1 ± 0.37	0.1 ± 0.35	n.s.
Number of follicles	8.2 ± 3.82	8.0 ± 3.79	7.5 ± 3.81	7.8 ± 3.71	n.s.
Embryo score of the transferred best embryo	2.4 ± 0.71	2.5 ± 0.62	2.4 ± 0.69	2.5 ± 0.72	n.s.
Day of embryo transfer	4.2 ± 0.83	4.2 ± 0.82	4.3 ± 0.78	4.2 ± 0.77	n.s.
Number of embryos transferred per patient	1.7 ± 0.52	1.7 ± 0.52	1.6 ± 0.55	1.7 ± 0.56	n.s.
Total number of transferred embryos	442	419	411	423	
Total number of embryos implanted	73	85	66	75	
Implantation rate (%)	16.5	20.3	16.0	17.7	
Embryo score of the 1st embryo	2.4 ± 0.71	2.5 ± 0.62	2.4 ± 0.69	2.5 ± 0.72	n.s.
Embryo score of the 2nd embryo	2.0 ± 0.77	2.0 ± 0.75	2.0 ± 0.72	2.1 ± 0.70	n.s.
Number of double embryo transfers	173	164	155	160	
Embryo score of the 3rd embryo	1.6 ± 0.67	2.0 ± 0.82	1.4 ± 0.53	1.7 ± 0.48	n.s.
Number of triple embryo transfers	12	7	9	13	
ENDOV (mm)	10.0 ± 2.00 (*n* = 253)	10.2 ± 2.07 (*n* = 245)	9.9 ± 2.00 (*n* = 240)	10.2 ± 2.02 (*n* = 244)	n.s.
ENDPU (mm)	10.4 ± 2.11 (*n* = 232)	10.8 ± 2.01 (*n* = 224)	10.4 ± 2.08 (*n* = 224)	10.6 ± 2.02 (*n* = 228)	n.s.
ENDET (mm)	11.4 ± 2.38 (*n* = 253)	11.8 ± 2.63 (*n* = 247)	11.6 ± 2.57 (*n* = 246)	11.4 ± 2.25 (*n* = 247)	n.s.
**Paternal Side Parameters**					
Paternal age (years)	38.3 ± 5.91 (*n* = 253)	38.3 ± 5.81 (*n* = 244)	38.6 ± 6.53 (*n* = 244)	38.4 ± 6.36 (*n* = 245)	n.s.
Sperm concentration (×10^6^/mL)	43.9 ± 39.88	45.3 ± 36.38	46.8 ± 42.02	50.3 ± 44.69	n.s.
Sperm motility (%)	41.8 ± 19.13	43.2 ± 18.07	42.9 ± 18.65	43.9 ± 19.96	n.s.
Oligospermia (*n*; %)	84 (33)	67 (27)	74 (30)	73 (30)	n.s.
Asthenozoospermia (*n*; %)	124 (49)	114 (46)	121 (49)	109 (44)	n.s.
Teratozoospermia (*n*; %)	100 (39)	88 (36)	87 (35)	83 (34)	n.s.
Normospermia (*n*; %)	99 (39)	103 (42)	103 (42)	109 (44)	n.s.
**Outcomes**					
Pregnant patients (*n*; %)	66 (26)	80 (32)	60 (24)	67 (27)	n.s.
Non-pregnant patients (*n*; %)	189 (74)	167 (68)	187 (76)	180 (73)	n.s.
Live birth (*n*; %)	27 (11)	34 (14)	22 (9)	23 (9)	n.s.

The data are presented as mean ± SD or number and percentage (*n*, %); n.s., not significant; *, *p*-value < 0.05; ***, *p* < 0.001. The clinical data were grouped to subcategories marked with bold letters. AMH, anti-Müllerian hormone; BMI, body mass index; ENDET, endometrial thickness at the time of embryo transfer; ENDOV, endometrial thickness on the day of triggering human chorionic gonadotropin injection; ENDPU, endometrial thickness on the day of puncture; FSH, follicle-stimulating hormone; GnRH, gonadotropin-releasing hormone; IUI, intrauterine insemination; LH, luteinizing hormone; PCOS, polycystic ovary syndrome; Sig., significance; TSH, thyroid-stimulating hormone; Q1–Q4, categories of quartiles examined.

## Data Availability

The data can be made available by the corresponding author on request.

## Supplementary Materials

The following supporting information can be downloaded at: https://www.mdpi.com/article/10.3390/jcm14134407/s1. Table S1: Embryo scoring system; Table S2: Randomset; Table S3: Correlation; Figure S1: (A) The distribution of TSH in different BMI groups and age groups. BMI and age value range is provided in the header with number of elements in each category. (B) Hexbinplot indicates the results of sensitivity analyses comparing normalized TSH, BMI values and pregnancy outcomes. The larger chart represent BMI compared to TSH, meanwhile the smaller plots show the effect of BMI and TSH data on pregnancy; Figure S2: Heatmap represents the Pearson correlation of the investigated parameters. References [20,21] are cited in the Supplementary Materials.
