# Supplementary material for: The Influence of Pre-IVF Day 2 TSH Levels on Treatment Success and Obstetric Outcomes: A Retrospective Single-Center Analysis with Machine Learning-Based Data Evaluation
Source: J Clin Med. 2025 Jun 20;14(13):4407. doi: 10.3390/jcm14134407 (PMC12250441; doi:10.3390/jcm14134407)
Supplement: Supplementary file 1 [file jcm-14-04407-s001.zip › TSH cikk supplementary material_EV.pdf]

The impact of TSH levels measured prior to in vitro fertilization on treatment success and obstetric outcomes: a single-center retrospective data analysis

Supplementary Materials

Methods

FSH treatment started on the 2nd–3rd day of menstruation, and it was adjusted according to patient response, who was monitored by serial transvaginal sonography and estrogen (E2) level from day five and every 2–3 days on. Once the leading follicles got to the diameter of 14 mm, 0.25 mg of GnRH antagonist was given daily until the day of trigger. Triggering was performed by hCG, when the patient had at least three follicles that were ≥18 mm. 36 hours after the hCG (Ovitrelle®, Merck) triggering, the oocytes were removed by transvaginal oocyte retrieval. Laboratory and transfer procedures were similar in all cycles and followed general laboratory protocols. The follicular fluid was collected in preheated round bottom 14 ml tubes (Thermo Fisher Scientific, Denmark) and held in a heating block calibrated at 37°C. The oocyte search was performed under laminar flow using 90 mm petri dishes (Thermo Fisher Scientific, Denmark). The cumulus-oocyte complexes were collected in Nunc IVF Center Well Dish (Thermo Fisher Scientific, Denmark) in G-MOPS PLUS (Vitrolife, Sweden) medium. Subsequently, the complexes were placed in 5-well dishes (Vitrolife, Sweden) in G-IVF (Vitrolife, Sweden) under oil overlay (Hypure Heavy Ovoil, Kitazato, Japan) in incubator with conditions set at 37°C, 6% CO_2 and 5% O_2 (K-Systems G210 InviCell, CooperSurgical, Denmark and PLANER Benchtop Incubator BT37, CooperSurgical, Denmark). Fertilization was performed 2-4 hours after oocyte retrieval, either through conventional IVF or through ICSI, depending predominantly on the semen parameters. For conventional IVF, semen with normal quality (normozoospermia) was applied to fertilize cumulus–oocyte complexes distributed in the wells of 5-well dishes in G-IVF media under oil overlay. Oocytes were denuded using hyaluronidase enzyme (80 IU/ml; SynVitro Hyadase, Origio, Denmark) for ICSI. Following fertilization, they were incubated in G1 and G2 (Vitrolife, Sweden) media in a 5-well dishes (Vitrolife, Sweden) in an incubator with conditions of 6% CO_2, 5% O_2 and 37°C (K-Systems G210 InviCell, CooperSurgical, Denmark, PLANER Benchtop Incubator BT37, CooperSurgical, Denmark). At 17–20 hours after successful insemination, zygotes were examined for the presence of two pronuclei including the paternal and the maternal pronucleus. If more than two pronuclei were observed in a zygote, it was classified as an abnormal zygote and discarded. For transfer, the best quality embryos at the cleavage- or blastocyst-stage were selected. However, only blastocyst-stage embryos were chosen for freezing on culturing day 5 or 6. Embryos were qualified based on number of blastomeres and percentage of fragmentation (17). Before embryo transfer, blastocysts were placed in EmbryoGlue (Vitrolife, Sweden) for 20-40 minutes. The embryos were transferred intrauterine with the Wallace Embryo Replacement Catheter (soft, 18 cm, CooperSurgical, USA). Two-dimensional transvaginal ultrasonography (Samsung Medison HS50; endocavitary probe: EVN4-9, 4-9 MHz) was utilized for follicular punctures and embryo transfers.

Supplementary Table S1. Embryo scoring system.

Day of culturing/Quality of embryo	Excellent quality (3 points)	Good quality (2 points)	Poor quality (1 point)
**Day 3**	8A1, 8B1, 10A1, 10B1	6A1, 6B1, 8A2, 6A2, 10A2, 12A1	8B2, 10B2, 6B2, 4A1, 2A1 or worse quality
**Day 4**	compact morula, early blastocyst	10A1, 10B1, 10A2, 12A1, early morula	8A1, 10B2 or worse quality
**Day 5**	1AA, 2AA, 3AA, 4AA, 5AA, 6AA	1BB, 1BA, 1AB, 2BB, 2AB, 2BA, 3BB, 3BA, 3AB, 4BB, 4BA, 4AB, early blastocyst, compact morula	1CC, 1BC, 1CB, 2CC, 2CB, 2BC, 3CC, 3CB, 3BC, 4CC, 4BC, 4CB, early morula, 12A1 or worse quality

Supplementary Table S1. *Embryo scoring system combined and modified after Gardner et al. (21) and Irani et al.(22); expansion status is described on a numerical scale from 1-6, while trophectoderm and inner cell mass are both categorized using letter A-B-C.*

The quality of embryos transferred is a key factor determining the success or failure of IVF treatment cycles. The detailed morphological embryo grading system by Gardner et al. (21) reliably represents the embryo quality and provides standardization in the assessment. The integration of embryo quality into statistical analysis is a major requirement for evaluating the outcome of IVF treatment cycles. To advance both research and clinical processes, the expansion status is described on a numerical scale from 1-6, while trophectoderm and inner cell mass are both categorized using letter A-B-C. The combinations of these variables result in 54 possible categorical embryo grades. To simplify the analysis and reduce the number of categorical variables, the embryo grade subgroups were clustered into three groups. Clustering a large number of embryo grades into classifications such as 'excellent, good, or poor' may lead to some data loss, but it enchases the interpretability of the analysis (22).

21. Gardner, D.K.; Weisssmann, A.; Howles, C.M.; Shoham, Z. *Textbook of Assisted Reproductive Techniques: Laboratory and Clinical Perspectives*, 2nd ed.; CRC Press: Boca Raton, FL, USA, 2004.

22. Irani, M.; Reichman, D.; Robles, A.; Melnick, A.; Davis, O.; Zaninovic, N.; Xu, K.; Rosenwaks, Z. Morphologic grading of euploid blastocysts influences implantation and ongoing pregnancy rates. *Fertil. Steril.* **2017**, *107*, 664–670. https://doi.org/10.1016/j.fertnstert.2016.11.012.

Supplementary Figure Legends

Supplementary Figure S1.

A) The distribution of TSH in different BMI groups and age groups. BMI and age value range is provided in the header with number of elements in each category. B) Hexbinplot indicates the results of sensitivity analyses comparing normalized TSH, BMI values and pregnancy outcomes. The larger chart represent BMI compared to TSH, meanwhile the smaller plots show the effect of BMI and TSH data on pregnancy.

Supplementary Figure S2. Heatmap represents the Pearson correlation of the investigated parameters.
