# Supplementary figures and images for: The Influence of Pre-IVF Day 2 TSH Levels on Treatment Success and Obstetric Outcomes: A Retrospective Single-Center Analysis with Machine Learning-Based Data Evaluation
Source: J Clin Med. 2025 Jun 20;14(13):4407. doi: 10.3390/jcm14134407 (PMC12250441; doi:10.3390/jcm14134407)

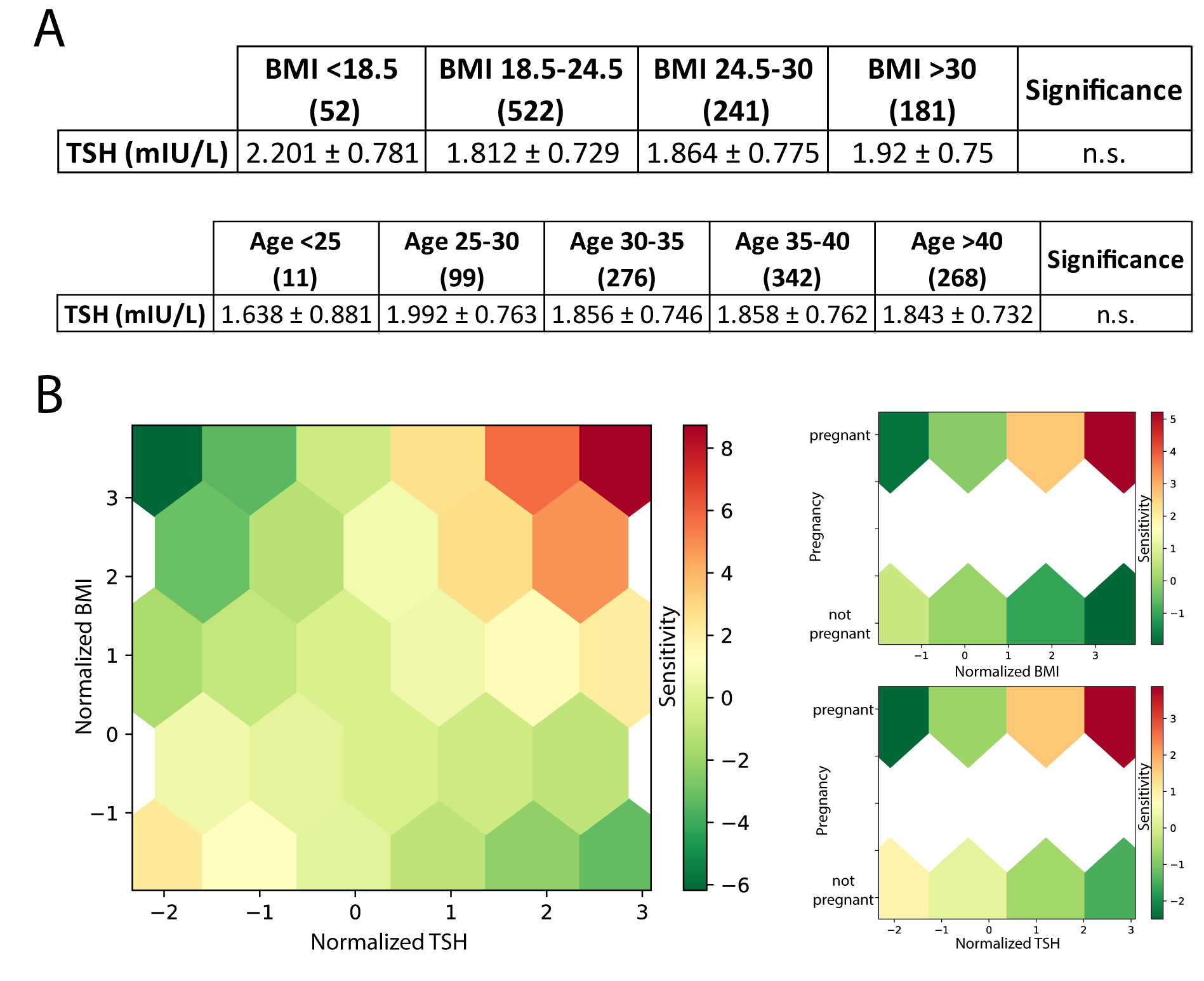

Supplement: Supplementary file 1 [file jcm-14-04407-s001.zip › Supplementary Figure S1_sensitivity.tiff]

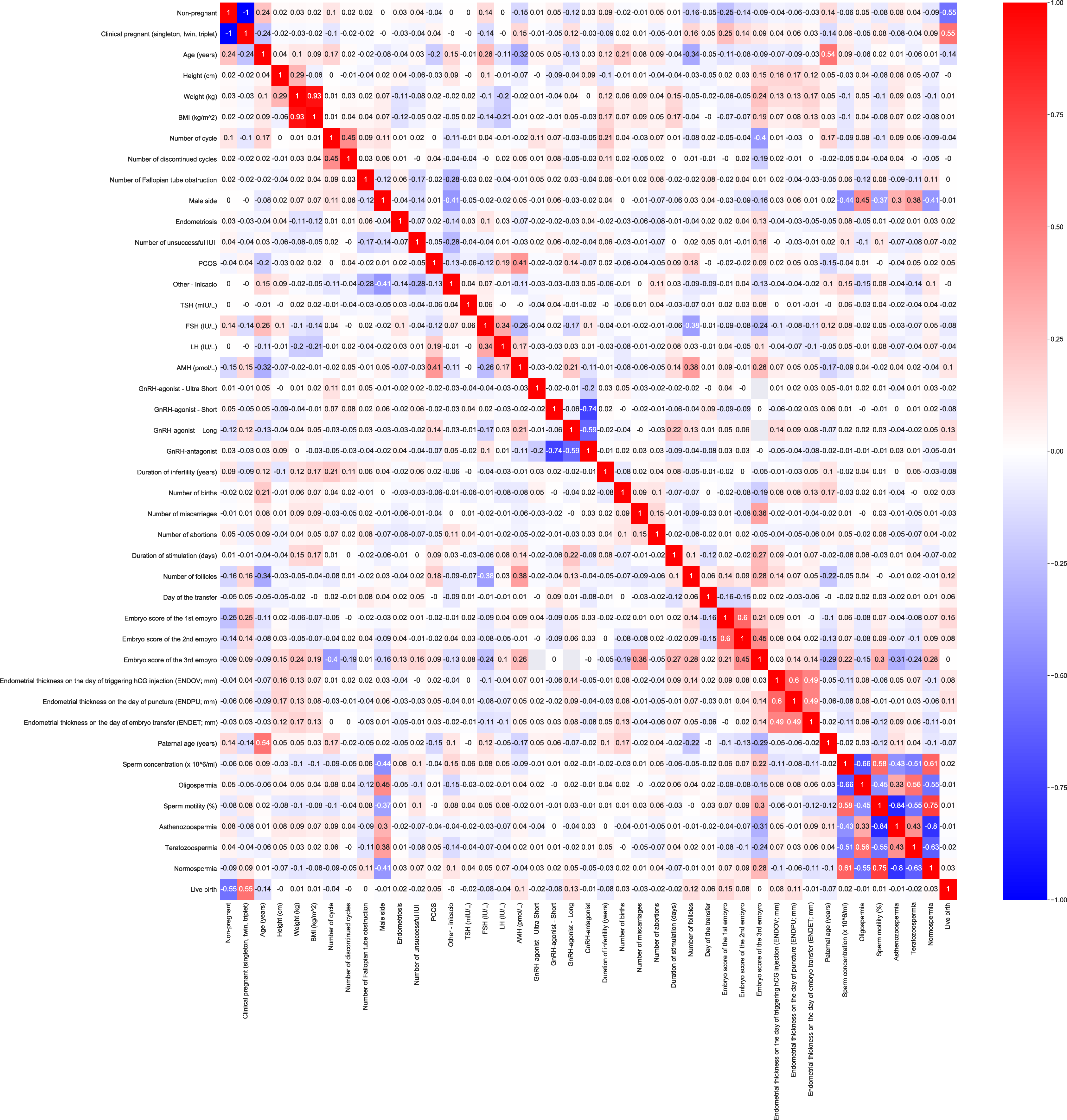

Supplement: Supplementary file 1 [file jcm-14-04407-s001.zip › Supplementary Figure S2_correlation.tiff]
